# The Relationship between Ciprofloxacin Resistance and Genotypic Changes in *S. aureus* Ocular Isolates

**DOI:** 10.3390/pathogens11111354

**Published:** 2022-11-15

**Authors:** Madeeha Afzal, Ajay Kumar Vijay, Fiona Stapleton, Mark Willcox

**Affiliations:** School of Optometry and Vision Sciences, University of New South Wales, Sydney, NSW 2041, Australia

**Keywords:** *Staphylococcus aureus*, genome sequences, ocular conditions, ciprofloxacin resistance phenotype, genetic mutations

## Abstract

*Staphylococcus aureus* (*S. aureus*) is a frequent cause of eye infections with some isolates exhibiting increased antimicrobial resistance to commonly prescribed antibiotics. The increasing resistance of ocular *S. aureus* to ciprofloxacin is a serious concern as it is a commonly used as a first line antibiotic to treat *S. aureus* keratitis. This study aimed to analyse genetic mutations in the genomes of 25 *S. aureus* isolates from infections or non-infectious ocular conditions from the USA and Australia and their relationship to ciprofloxacin resistance. Overall, 14/25 isolates were phenotypically resistant to ciprofloxacin. All isolates were analyzed for mutations in their quinolone resistance-determining regions (QRDRs) and efflux pump genes. Of the fourteen resistant isolates, 9/14 had ciprofloxacin resistance mutations within their QRDRs, at codons 80 or 84 within the *parC* subunit and codon 84 within the *gyrA* subunit of DNA gyrase. The highest resistance (MIC = 2560 μg/mL) was associated with two SNPs in both *gyrA* and *parC*. Other resistant isolates (3/14) had mutations within *norB.* Mutations in genes of other efflux pumps and their regulator (*norA*, *norC*, *mepA*, *mdeA*, *sepA*, *sdrM*, *mepR*, *arlR*, and *arlS*) or the DNA mismatch repair (MMR) system (*mutL* and *mutS*) were not associated with increased resistance to ciprofloxacin. The functional mutations associated with ciprofloxacin resistance in QRDRs (*gyrA* and *parC*) and *norB* suggests that these are the most common reasons for ciprofloxacin resistance in ocular isolates. Novel SNPs of *gyrA* Glu-88-Leu, Asn-860-Thr and Thr-845-Ala and IIe-855-Met, identified in this study, need further gene knock out/in studies to better understand their effect on ciprofloxacin resistance.

## 1. Introduction

*Staphylococcus aureus* (*S. aureus*) is a highly adaptable opportunistic pathogen renowned for its ability to evade the immune system and cause a variety of infections [1]. Approximately 30% of humans are colonized with *S. aureus* [2]. It can cause a range of ocular diseases in humans, including infectious conjunctivitis, sight-threatening microbial keratitis (MK) and non-infectious corneal infiltrative events (niCIE) [3,4,5,6].

*S. aureus* is naturally resistant to many antimicrobials and can acquire resistance through horizontal gene transfer or mutations in chromosomal genes [7]. Increasing antimicrobial resistance of *S. aureus* has been identified as a public health threat by the World Health Organization [8]. Eye infections with multidrug resistant *S. aureus* are difficult to treat [9]. Fluoroquinolones are broad spectrum and widely prescribed antibiotics used to treat staphylococcal ocular infections [10]. Ciprofloxacin is commonly prescribed as a monotherapy for ocular infections [11]. However, increased resistance of fluoroquinolones in *S. aureus* has been reported [7,12,13,14], possibly due to their excessive use [15]. Whilst ocular microbial keratitis isolates from Australia were reported to be mostly (93–100%) susceptible to ciprofloxacin [16,17,18,19], a surveillance study from USA found that 36% of the ocular *S. aureus* isolates were resistant to ciprofloxacin [20]. Previous phenotypic data of the isolates used in the current study showed that some strains were resistant to ciprofloxacin [21].

The mechanisms of fluoroquinolone resistance in *S. aureus* from non-ocular infections have been previously studied and the most common mechanism of fluoroquinolone resistance is due to mutations in the genes that encode primary and secondary target sites of the fluoroquinolones in topoisomerase IV (*parC*/*parE*) and DNA gyrase (*gyrA*/*gyrB*) [22,23] and the efflux of the antibiotics from the cells [24,25]. Over expression of the genes *norA* and *norB* that encode for efflux pumps can reduce susceptibility to fluoroquinolones [25,26,27]. Another multidrug efflux pump, *norC*, when overexpressed can cause low-level fluoroquinolone resistance [28]. The expression of *norA*, *norB* and *norC* is regulated by *mgrA* [29], which therefore plays a role in modulating resistance to fluoroquinolones [27,28,30]. Other efflux pumps encoded by *mepA* [31,32], *mdeA* [33,34], *sepA* [35], *sdrM* [36], and *mepR* [37], can also confer resistance to fluoroquinolones. The two-component regulatory system *arlS*/*arlR*, which modifies the expression of *norA*, can mediate resistance to fluoroquinolones [38].

Topoisomerase IV is the primary target of fluoroquinolones in *S. aureus*, and mutation in *parC* is often the first step in the resistance to most fluoroquinolones [39,40,41]. Resistant mutants have changes to the quinolone resistance-determining regions (QRDRs) located in A and C subunits of topoisomerase IV and DNA gyrase, encoded by *parC* and *gyrA*, respectively. Combinations of single point mutations within *gyrA* such as Glu-88-Gly plus Ser-84-Leu (MIC = 200 μg/mL) are associated with higher ciprofloxacin MICs than single point mutations (e.g., Glu-88-Gly MIC = 12.5 μg/mL) [42]. Similarly, two combinations of single point mutations within *parC*, Glu-84-Val or Ala-48-Thr in combination with Ser-80-Phe, have been associated with higher ciprofloxacin MICs (range 64–256) than only the single point Ser-80-Phe mutation (range 8–128) [43]. There is a stepwise emergence of ciprofloxacin resistance in *S. aureus*, which first involves *parC* mutations conferring low-level resistance, followed by *gyrA* mutations leading to higher level resistance (range 64–256) [40,44].

The frequency of mutation is important to consider in fluoroquinolone resistant *S. aureus* isolates. Mutation rates are higher in the strains that carry mutations in the DNA mismatch repair (MMR) system and increased mutations in the MMR system can lead to high-levels of fluoroquinolones resistance [45,46,47,48]. The DNA mismatch repair system in *S. aureus* is based on MutS-MutL and functions to correct errors and preserve the integrity of the genome [49]. Normally, mismatches that occur during DNA replication are repaired by this mismatch repair system, but certain mutations in *mutS* and *mutL* can lead to the rapid accumulation of mutations in newly synthesized DNA strands [46]. Mutations in the MMR system can lead to the development of hypermutations in isolates. Strong mutators have defects in their MMR system with mutations predominantly in *mutS* [50]. For *S. aureus*, little is known about the genetic basis of hypermutability. However, inactivation of *mutS* in *S. aureus* led to the hypermutator phenotype [51] and hypermutable *S. aureus* might exist in naturally occurring populations [52,53]. A study detected a high proportion of hypermutable *S. aureus* strains isolated from cystic fibrosis (CF) patients [48]. In many cases, hypermutability has been related to defects in the *mutS* and *mutL* of the MMR system [54]. Gram positive bacteria have an MMR system that is functionally equivalent to that of *E. coli*, although the *mutH* component of MMR has not been found in *S. aureus.* Mutations in *mutS* and *mutL* reduce the ability of the bacterium to repair DNA lesions [55] and so may be associated with increased numbers of SNPs or other genetic changes.

The current study aimed to examine the relationship between the phenotype of ciprofloxacin resistance and mutations in fluoroquinolone resistance and MMR genes in *S. aureus* ocular isolates (including those from infectious conjunctivitis, sight-threatening microbial keratitis (MK) and non-infectious corneal infiltrative events, niCIE) from the USA and Australia.

## 2. Materials and Methods

Twenty-five *S. aureus* ocular isolates, 19 from infections (MK or conjunctivitis) and six isolates from niCIEs were examined (Table 1). These isolates were selected from a larger collection of strains based on their published susceptibility to ciprofloxacin and possession of virulence [56] and acquired resistance genes [57].

Susceptibility of *S. aureus* isolates to ciprofloxacin (Sigma-Aldrich, Inc., St. Louis, MO, USA) had been investigated in a previous study using the broth microdilution method following the protocol of the Clinical and Laboratory Standard Institute [58]. The lowest concentration of an antibiotic in which no noticeable growth (turbidity) observed was taken as the average minimum inhibitory concentration (MIC) from three replicates and the break point was established according to the published standards.

Bacteria were revived from frozen stock in tryptone soya broth (TSB; Oxoid Ltd., Basingstoke, UK). Bacterial DNA from each *S. aureus* strain was extracted using QIAGEN DNeasy blood and tissue extraction kit (Qiagen, Hilden, Germany) as per the manufacturer’s instructions. The extracted DNA was quantified and its purity-checked using Nanodrop (NanoDrop Technologies, Wilmington, DE, USA), Qubit fluorometer (Life Technologies, Carlsbad, CA, USA) and 1% agarose gel electrophoresis. The extracted DNA was dried for transport to the sequencing facility at Singapore Centre for Environmental Life Sciences Engineering, Singapore. The Nextera XT DNA library preparation kit (Illumina, San Diego, CA, USA) was used to prepare paired-end libraries. All the libraries were multiplexed on one MiSeq run.

The quality of raw reads was analysed using online tool FastQC version 0.117 (https://www.bioinformatics.babraham.ac.uk/projects/fastqc, accessed on 9 July 2021). Trimmomatic version 0.38 (http://www.usadellab.org/cms/?page=trimmomatic, accessed on 9 July 2021) was used for trimming the adapters from the reads with quality and length filtering (SLIDINGWINDOW:4: 15 MINLEN:36) [59]. The reads were de novo assembled using Spades v3.15.0 with program’s default setting [60] and the standard *S. aureus* strain NCTC 8325 was re-assembled using Spades to avoid assembly introduced errors. Genomes were annotated using Prokka v1.12 (https://github.com/tseemann/prokka, created by Torsten Seemann, Victoria, Melbourne, Australia, accessed on 20 October 2021) with the GeneBank^®^ compliance flag [61]. The genome of *S. aureus* NCTC 8325 (NC_007795.1) (reference strain in this study) was re-annotated with Prokka to avoid annotation bias. To identify mutations in the QRDRs (*gyrA*, *gyrB*, *parC* and *parE*), efflux pump genes (*norA*, *norB*, *norC*, *mgrA*, *mepA*, *mdeA*, *sepA*, *sdrM*, and *mepR*) their regulators (*arlR* and *arlS*) and MMR genes (*mutL* and *mutS*), the genome sequences were analysed using Snippy v4.2 (https://github.com/tseemann/snippy, created by Torsten Seemann, Victoria, Melbourne, Australia, accessed on 21 November 2021) with the program’s default settings and compared with reference genome *S. aureus* NCTC 8325 (NC_007795.1). The non-synonymous mutations were further assessed for amino acid substitution and the effect on protein function using SIFT [62].

## 3. Results

### 3.1. Non-Synonymous Variations in the Genes of the Ocular Isolates

Several types of non-synonymous variations were found in the core genomes of the *S. aureus* isolates (Supplementary Table S1). These non-synonymous mutations included single nucleotide polymorphisms (SNPs), multi-nucleotide polymorphisms (MNPs), deletions, insertions and complex variations (where more than one change occurred at one specific location compared to the reference strain NCTC 8325). The total variations in the isolates ranged from 847 in SA32 to 44,256 in SA34. There was a median of 17,879 (IQR = 15,853–29,471) variations in genomes of infectious strains and a median of 25,612 (IQR = 39,481–17,435) variations in niCIE strains from Australia. Similarly, there was median of 17,836 (IQR = 2339–18,157) variations in genomes of infectious strains from the USA. Isolate SA34, a ciprofloxacin susceptible strain, had the highest number of variations (44,256) and SNPs (34,517) and isolate SA32, another ciprofloxacin susceptible strain, had the lowest number of variations (847) and SNPs (719). There were no significant differences (Mann Whitney U-test) between any type of variant for any ocular condition or country.

### 3.2. Genetic Variations in Quinolone-Associated Genes

Phenotypically, 56% (14/25) of isolates were resistant to ciprofloxacin. The Resfinder database was used to find antibiotic resistance genes of *S. aureus* isolates. The number of non-synonymous SNPs in the quinolone-resistance-determining regions (QRDRs; *gyrA*, *gyrB*, *parC* and *parE*), and efflux pump genes (*norA*, *norB*, *norC*, *mgrA*, *mepA*, *mdeA*, *sepA*, *sdrM*, *mepR*, *arlR* and *arlS*) are shown in Supplementary Table S2. Most of the SNPs were found in *norB*, with a median of 12 SNPs per strain, followed by *mepA* with a median of 3 SNPs per strain (Supplementary Table S2). No non-synonymous SNPs were found in *norA*, *norC*, *mdeA*, *mepR* and *arlR*. Only one SNP was found in *sepA* (in resistant strain M43-01), but this was not classified as a functional mutation. Non-functional SNPs were also found in *sdrM*, two in resistant strain M43-01, one in resistant strain M71-01 (both infectious strains from Australia) and two in susceptible strain SA20 (niCIE), and *arlS*, giving a median of zero in resistant isolates and one in susceptible isolates (Supplementary Table S2).

The mutations that caused changes in the amino acid sequence (functional mutations; Table 1) were further compared to the previously published ciprofloxacin susceptibility of these isolates. Table 1 only shows those mutations (SNPs) within *gyrA*, *parC*, *norB* and *mgrA*, which caused changes in amino acid sequences (functional mutations). All other mutations that would be unlikely to have a functional effect on ciprofloxacin susceptibility (as the genetic variation did not result in amino acid changes) were excluded from further analysis.

There were no SNPs that caused functional changes in fluoroquinolone resistant genes in any of the ciprofloxacin susceptible isolates (Table 1). Mutations in *gyrA* or *parC* that caused changes in amino acids were found in 64% (9/14) of ciprofloxacin resistant strains with MICs ranging from 32–2560 μg/mL. Strain SA112 (infectious USA strain) that had the highest MIC (2560 μg/mL) was the only strain to have two non-synonymous SNPs that would likely have resulted in functional changes to the proteins in both *gyrA* and *parC* (Table 1), as well as a non-synonymous SNP in *parC* (Asp-796-Asn; Supplementary Table S2) that, whilst not likely to have resulted in a functional change, was only present in this isolate. SA112 (infectious USA strain) also had a non-synonymous SNP in *norB* (Val-189-Ile) that was unlikely to have had a functional impact on the protein but was shared only by the strains SA111 and SA113 (infectious USA strains) that had the next highest MICs (1280 μg/mL). Strains SA111 and SA113 also had one SNP in each *gyrA* and *parC* that would likely have resulted in a functional change in the proteins (Table 1), as well as being the only strains with the SNP Leu-140-Ile in *norB*, that was predicted to have a functional impact on NorB.

The two infectious strains that had an MIC of 128 μg/mL, SA101 (USA) and M43-01 (Australian), had SNP Ser-84-Leu in *gyrA* and either Ser-80-Tyr or Ser-80-Phe in parC, but were otherwise divergent in the possession of SNPs in these genes and in norB, mepA, sepA, srdM and arlS (Table 1 and Supplementary Table S2), although most of the SNPs in these genes were not predicted to have a functional impact on the proteins. The three infectious strains with MIC of 64 μg/mL had different SNP profiles (Table 1 and Supplementary Table S2) with SNPs predicted to have functional consequences in *gyrA* and norB only (SA107, from USA), *gyrA* and *parC* only (SA90 from USA) or norB only (M5-01 from Australia). The two infectious strains SA102 and SA103 (from USA) with MIC 32 μg/mL had almost identical SNPs which occurred in only *gyrA*, *parC* and *norB*, with the exception of Phe-521-Tyr or Glu-422-Asp in parC (Table 1 and Supplementary Table S2). This combination of SNPs in *gyrA*, *parC* and *norB* was shared with infectious strain SA101 from USA, with the exception that SA101 did not have a second SNP in *parC*.

Infectious strain SA114 (USA) with an MIC of 8 μg/mL had only one SNP with a predicted effect on protein function. This occurred in *norB* Arg-168-Cys (Table 1). There were three strains, M71-01, SA136 (infectious Australian) and SA31 (niCIE), that had MIC 4 μg/mL. These had very different SNP profiles, with only strain SA136 having any SNPs that were predicted to have functional consequences, and these all occurred in *norB*. SA31 did not contain any SNPs in any gene that did not also occur in ciprofloxacin susceptible isolates.

Mutations in norB gene were commonly found in the ocular S. aureus isolates. Overall, 98 different types of mutations were found in norB, and out of these, 23 caused changes in the amino acid sequence. Seven mutations of norB, Leu-412-Ile, Tyr-289-Phe, Leu-140-Ile, Ile-12-Tyr, Ser-331-Tyr, Ala-186-Tyr, and Agr-168-Cys were only found in 36% (5/14) of ciprofloxacin resistant strains (Table 1), 15 mutations were found in both ciprofloxacin resistant and susceptible isolates and one mutation Ser-407-Ala was found in all 25 isolates.

In the current study, only one ciprofloxacin resistant infectious isolate from USA (SA113) had a functional mutation in *mgrA*, but this isolate also had mutations in *gyrA* and *parC*. As that isolate had the same MIC as another isolate without a mutation is *mgrA***,** its relationship to ciprofloxacin resistance remains uncertain in ocular isolates.

### 3.3. Mutations in the DNA Mismatch Repair System

This study also examined mutations in the genes involved in the DNA mismatch repair system (MMR). The mutations in the MMR system included SNPs, indels and complex variants. The number of mutations in mutL ranged from 0 to 9 and mutations in mutS ranged from 1 to 11. Details of mutations occurring in amino acid sequences are provided in Supplementary Table S3. The mutations in mutL and mutS which were predicted to have caused changes in the amino acid sequences in ciprofloxacin resistant or susceptible strains are shown in Table 2. These mutations were distributed between the ciprofloxacin resistant or susceptible strains and there was no relationship between possession of any type of mutation and SNPs in mutL or mutS.

## 4. Discussion

The study investigated non-synonymous mutations in 25 *S. aureus* isolates from infectious (MK and conjunctivitis) and non-infectious (niCIE) ocular conditions from USA and Australia. Based on previous phenotypic susceptibility studies, it was expected that there would be genetic changes that resulted in resistance to ciprofloxacin, such as mutations in quinolone resistance-determining regions (QRDRs: *gyrA*, *gyrB*, *parC* and *parE*) or efflux pumps (*norA*, *norB*, *norC*, *mgrA*, *mepA*, *mdeA*, *sepA*, *sdrM*, and *mepR*) and this study aimed to gain further insight into the impact of these mutations on ciprofloxacin MIC. The current study replicated the results of the previous study [21] of the same isolates that showed that resistance to ciprofloxacin was more common in isolates recovered from ocular infections compared to non-infectious corneal infiltrative events.

Two non-synonymous SNPs that would likely have resulted in functional changes to the proteins in both *gyrA* (Ser-84-Leu, Glu-88-Lys) and *parC* (Ser-80-Tyr, Glu-84-Lys; Table 1) and a non-synonymous SNP in *parC* (Asp-796-Asn; Supplementary Table S2) that was not predicted to have resulted in a functional change, were only present in the isolate (SA112; USA infectious strain) with the highest MIC (2560 μg/mL). A previous study on ocular isolates of *S. aureus* reported that strains containing *gyrA* Ser-84-Leu plus Glu-88-Lys/Ala with *parC* Ser-80-Tyr plus Glu-84-Gly (or Ser-80-Tyr alone) had MIC against ciprofloxacin of 256 μg/mL [63]. Most (6/8) non-ocular isolates of *S. aureus* with *gyrA* Ser-84-Leu plus Glu-88-Lys/Ala with *parC* Ser-80-Tyr plus Glu-84-Gly had ciprofloxacin MIC of ≥256 [64]. Therefore, the extremely high MIC for strain 112 (2560 μg/mL) may have been produced by the novel combination of mutations found in the current study in these genes, *gyrA* Ser-84-Leu plus Glu-88-Leu with *parC* Ser-80-Tyr plus glu-88-Lys. It is possible that these SNPs in combination with Asp-796-Asn in *parC*, had a role in the high level of resistance. The USA infectious strains SA111 and SA113 with ciprofloxacin MIC of 1280 μg/mL had a single SNP resulting in presumed functional changes in the gene product of *gyrA* (Ser-84-Leu) and a SNP in *parC* (Ser-80-Tyr). The combination of these SNPs has been shown to be associated with MICs of ≥256 μg/mL for ciprofloxacin in other ocular isolates [65] and also non-ocular isolates of *S. aureus* [65].

Two mutations of *gyrA* that were predicted to affect the function of the protein, Asn-860-Thr and Thr-845-Ala, in combination with IIe-855-Met, were found in the current study to be associated with strain M43-01 that had a relatively high MIC of 128 μg/mL. These mutations have not been reported previously, and their effect of the proteins function should be studied. Mutations in *gyrA* (Asn-860-Thr) in combination with *norB* (Leu-412-IIe and Tyr-289-Phe) may have caused the MIC of 64 μg/mL of strain SA107. However, the only functional mutations in strain M5-01 with an MIC of 64 μg/mL that occurred in *norB* (IIe-12-Thr, Ser-331-Thr and Ala-186-Thr) were also found in strain SA136 with an MIC of 4 μg/mL, and so these mutations may not have been the only ones to cause the higher MIC in strain M5-01.

Three types of *parC* mutations (Ser-80-Tyr, Ser-80-Phe, and Ser-80-Tyr in combination with Glu-84-Lys) responsible for quinolone resistance in the current study supports previous findings [44,64,66,67,68]. A previous study demonstrated that mutations in QRDR (*parC* and *gyrA*) confer resistance up to a certain level (8 to 32 μg/mL for ciprofloxacin), above which resistance is mainly driven by the efflux of the antibiotic [68]. The higher MIC for the ocular isolates in the current study may be associated with SNP Leu-140-Ile in the efflux pump *norB* that was only found in two infectious strains (SA112 and SA113). The SNPs in *norB* that affect its expression are not yet fully evaluated. One study has found that Met-314-Ile in *norB* may be associated with reduced function [69], but the effects of other SNPs are not yet known. It would be of interest in future studies to determine if strains with 2–4 QRDR in combination with efflux pump gene (*norB*) mutations have the high MIC values reported here and the effect of novel *gyrA* mutations Asn-860-Thr and Thr-845-Ala IIe-855-Met on MIC values.

One study also showed that efflux is an important contributor to fluoroquinolone resistance in *S. aureus* and suggests that it is as a major mechanism in the early stages of resistance development [68]. In the current study, one strain SA114 (infectious strain from Australia) with MIC = 8 μg/mL had mutation in *norB* (Arg-168-Cys), suggesting that this mutation of *norB* may have been responsible for resistance to ciprofloxacin. Additionally, two infectious strains, M5-01 and SA136 (from Australia), with an MIC of 64 μg/mL and 4 μg/mL, respectively, had the same mutations in *norB* (IIe-12-Thr in combination with Ser-331-Thr and Ala-186-Thr). The effect of these functional mutations of *norB* in ciprofloxacin resistant isolates showing different MIC values is uncertain, and gene knock out/knock in studies might help to better understand the role of mutations in *norB* and its effect on ciprofloxacin resistance level.

Mutations in *mutL* were observed in eight *S. aureus* isolates SA111, SA112, SA113, SA90, SA86, M71-01, SA25, and SA48. Three out of these eight isolates, SA86, SA27 and SA48, were phenotypically ciprofloxacin susceptible isolates and showed no QRDRs or efflux pump mutations associated with ciprofloxacin resistance. However, the other five isolates, SA111, SA112, SA113, SA114 and M71-01, were phenotypically ciprofloxacin resistant isolates, and showed mutations in QRDRs (*gyrA* and *parC*), except isolate SA114, which only showed mutation in efflux pump gene *norB*, and M71-01, which showed no mutations in QRDRs or the efflux pump. Mutations in *mutS* were observed in four *S. aureus* isolates SA129, SA27, SA114 and SA31 that suggests that these isolates are hypermutators. While SA129 and SA27 were ciprofloxacin susceptible isolates with no QRDRs and efflux mutations, SA114 and SA31 were ciprofloxacin resistant isolates with mutations in *mutS* and *norB*, but no mutations in QRDR. The current findings suggest that there is no relationship between mutations in ciprofloxacin resistance and MMR system and resistance to ciprofloxacin is related primarily to mutations in QRDRs (*gyrA* and *parC*), or efflux pump *norB*. Further in-depth studies are required to understand the influence of the MMR system on genomic changes. Additionally, as SNPs can be a result of poor sequencing quality, it is important to have a good sequencing depth at those positions to identify them as a mutation rather than sequencing error [70].

Two strains (M71-01 and SA31) that had low levels of resistance to ciprofloxacin (4 μg/mL) were not found to possess any SNPs predicted to change any of the functions of the products of the genes tested. This may mean that SNPs had effects on systems such as translation and transcription. Some SNPs might affect binding of transcription factors or translation machinery. These possible effects should be studied in future experiments, such as examining levels of mRNA for the genes and any association with the SNPs.

## 5. Conclusions

Ciprofloxacin resistant *S. aureus* tended to have mutations in QRDRs (*gyrA* and *parC*), with high-level resistance when both genes were mutated and some limited association with SNPs in the efflux pump *norB* that may contribute to ciprofloxacin resistance. Overall, these findings have extended our understanding of the relationship between ciprofloxacin resistance and their mutations in *S. aureus* infectious and non-infectious ocular conditions. These results indicate that antimicrobial stewardship is required when using topical antibiotic eye drops to treat keratitis and conjunctivitis. Further gene knock out/knock in isogeneic mutant studies will help to better understand the effects of novel SNPs on ciprofloxacin resistance.

## Figures and Tables

**Table 1:** MIC range in *S. aureus* isolates and functional mutations in QRDRs and efflux pump genes.

Strain	Ocular Condition	Ciprofloxacin Sensitivity	MIC (μg/mL) [21]	*gyrA*	*parC*	*norB*	*mgrA*
SA112	USA keratitis	Resistant	2560	Ser-84-Leu, Glu-88-Leu	Ser-80-Tyr, Glu-84-Lys	-	-
SA111	USA keratitis		1280	Ser-84-Leu	Ser-80-Tyr	Leu-140-IIe	-
SA113	USA keratitis		1280	Ser-84-Leu	Ser-80-Tyr	Leu-140-IIe	Leu-64-Pro
SA101	USA conjunctivitis		128	Ser-84-Leu	Ser-80-Tyr	-	-
M43-01	Australia keratitis		128	Ser-84-Leu, Thr-845-Ala, IIe-855-Met	Ser-80-Phe	-	-
SA107	USA keratitis		64	Asn-860-Thr	-	Leu-412-IIe Tyr-289-Phe	-
M5-01	Australia keratitis		64	-	-	IIe-12-Thr, Ser-331-Thr, Ala-186-Thr	-
SA90	USA conjunctivitis		64	Ser-84-Leu	Ser-80-Tyr Glu-84-Lys	-	-
SA102	USA conjunctivitis		32	Ser-84-Leu	Ser-80-Phe	-	-
SA103	USA conjunctivitis		32	Ser-84-Leu	Ser-80-Tyr	-	-
SA114	USA keratitis		8	-	-	Agr-168-Cys	-
M71-01	Australia keratitis		4	-	-	-	-
SA136	Australia conjunctivitis		4	-	-	IIe-12-Thr, Ser-331-Thr, Ala-186-Thr	-
SA31	niCIE		4	-	-	-	-
SA86	USA conjunctivitis	Susceptible	1				
SA34	Australia keratitis		1				
SA129	Australia keratitis		1				
M19-01	Australia keratitis		1				
M28-01	Australia keratitis		1				
SA46	Australia conjunctivitis		1				
SA20	niCIE		1				
SA25	niCIE		1				
SA27	niCIE		1				
SA32	niCIE		1				
SA48	niCIE		1				

SNPs analysis using *Staphylococcus aureus* NCTC 8325 as a reference strain, using the default parameters of Snippy v4.2, excluding SNPs identified in regions that had arisen by recombination. Quinolone resistance determining regions; QRDRs (*gyrA*, *parC*) and efflux pump genes (*norB*, *mgrA*).

**Table 2:** MMR gene mutations that were predicted to have an effect on protein function in ciprofloxacin susceptible and resistant strains.

*S. aureus* Isolates	Ciprofloxacin Sensitivity	CIP MIC (μg/mL)	MMR Genes and Sites of Mutations	
			*mutL*	*mutS*
SA112	Resistant	2560	His-347-Tyr	-
SA111		1280	His-347-Tyr	-
SA113		1280	His-347-Tyr	-
SA101		128	-	-
M43-01		128	-	-
SA107		64	-	-
M5-01		64	-	-
SA90		64	His-347-Tyr	-
SA102		32	-	-
SA103		32	-	-
SA114		8	-	Gln-531-His
M71-01		4	His-347-Tyr	-
SA136		4	-	-
SA31		4	-	Gln-531-His
SA86	Susceptible	1	His-347-Tyr	-
SA34		1	-	Ala-172-Val
SA129		1	-	Gln-531-His
M19-01		1	Val-583-IIe	-
M28-01		1	Val-583-IIe	-
SA46		1	-	-
SA20		1	-	-
SA25		1	His-347-Tyr	-
SA27		1	-	Gln-531-His
SA32		1	-	-
SA48		1	His-347-Tyr	-

## Data Availability

Data is contained within the article and available upon request.

## Supplementary Materials

The following supporting information can be downloaded at: https://www.mdpi.com/article/10.3390/pathogens11111354/s1, Table S1: Frequency of different types of variations in the genes of *S. aureus* isolates.; Table S2: Genes and sites of their mutations (all functional and non-functional mutations) of S. aureus ocular strains from infections and non-infectious disease group (there were no mutations detected in *nor A*, *norC*, *mdeA*, *mepR* or *arlR*); Table S3: Mutations in MMR system of *S. aureus* ocular strains from infections and non-infectious disease group.

## Nucleotide Accession

The nucleotide sequences are available in the Genebank under the Bio project accession number PRJNA859391, accession numbers; JANHMY000000000, JANHMZ000000000, JANHNA000000000, JANHNB000000000, JANHNC000000000, JANHND000000000, JANHNE000000000, JANHNF000000000, JANHNG000000000, JANHNH000000000, JANHNI000000000, JANHNK000000000, JANHNL000000000, JANHNM000000000, JANHNN000000000, JANHNO000000000, JANHNP000000000, JANHNQ000000000, JANHNR000000000, JANHNS000000000, JANHNT000000000, JANHNU000000000, JANHNV000000000, JANHNW000000000.
