# Supplementary material for: The Relationship between Ciprofloxacin Resistance and Genotypic Changes in S. aureus Ocular Isolates
Source: Pathogens. 2022 Nov 15;11(11):1354. doi: 10.3390/pathogens11111354 (PMC9695201; doi:10.3390/pathogens11111354)
Supplement: Supplementary file 1 [file pathogens-11-01354-s001.zip › pathogens-1944683-supplementary.pdf]

Supplementary Table S1. Frequency of different types of variations in the genes of *S. aureus* isolates.

Ocular condition	Country	*S. aureus* isolates	Total sequence length (bp)	Total variations	complex	insertions	deletions	MNPs	SNPs
Conjunctivitis	Australia	SA136	3,035,909	18,610	2,930	160	181	426	14,913
Conjunctivitis	Australia	SA46	2,903,724	17,879	2,221	155	224	448	14,831
Conjunctivitis	USA	SA90	3,015,554	18,049	2,208	148	216	458	15,019
Conjunctivitis	USA	SA86	4,579,417	18,025	2,136	154	226	426	15,083
Conjunctivitis	USA	SA101	3,602,977	2,339	337	33	71	47	1,851
Conjunctivitis	USA	SA103	3,069,147	2,267	322	33	70	49	1,793
Conjunctivitis	USA	SA102	3,406,253	1,367	136	37	68	27	1,099
		**Median**	**3,069,147**	**17,879**	**2,136**	**148**	**181**	**426**	**14,831**
Keratitis	Australia	SA34	2,914,342	44,256	7,904	275	317	1,246	34,517
Keratitis	Australia	SA129	3,105,791	40,332	6,428	270	330	1,105	32,199
Keratitis	Australia	M5-01	2,975,620	18,139	2,802	164	193	438	14,542
Keratitis	Australia	M28-01	2,960,866	16,238	2,054	149	204	401	13,430
Keratitis	Australia	M19-01	2,893,905	15,944	1,938	149	185	390	13,282
Keratitis	Australia	M43-01	3,029,867	15,762	1,883	129	177	389	13,184
Keratitis	Australia	M71-01	2,918,758	15,077	1,951	130	182	365	12,449
Keratitis	USA	SA112	3,170,760	18,320	2,217	154	217	466	15,266
Keratitis	USA	SA111	3,113,006	18,157	2,193	150	222	455	15,137
Keratitis	USA	SA113	3,014,859	17,647	2,159	146	212	432	14,698
Keratitis	USA	SA107	3,599,003	15,867	1,919	114	183	388	13,263
Keratitis	USA	SA114	3,175,242	39,301	6,224	263	328	1014	31,472
		**Median**	**3,022,363**	**17,893**	**2,176**	**150**	**208**	**435**	**14,620**
niCIE	Australia	SA31	2,976,006	40,473	6,495	264	330	1,127	32,257
niCIE	Australia	SA27	2,919,830	39,481	6,236	258	325	1,086	31,576
niCIE	Australia	SA20	2,909,603	33,525	4,642	270	328	895	27,390
niCIE	Australia	SA25	2,907,754	17,700	2,147	144	210	447	14,752
niCIE	Australia	SA48	2,922,947	17,435	2,127	146	222	224	14,495
niCIE	Australia	SA32	2,990,036	847	29	39	56	4	719
		**Median**	**2,921,388**	**25,613**	**3,395**	**202**	**274**	**671**	**21,071**

SNP = single nucleotide polymorphism, MNP = multi-nucleotide polymorphism, niCIE = non-infectious corneal infiltrative events

Supplementary Table S2. Genes and sites of their mutations (all functional and non-functional mutations) of *S. aureus* ocular strains from infections and non-infectious disease group (there were no mutations detected in *nor A, norC, mdeA, mepR* or *arlR*).

***S. aureus* isolates**	**CIP MIC (µg/ml)**	***gyrA***	***gyrB***	***parC***	***parE***	***norB***	***mgrA***	***mepA***	***sepA***	***sdrM***	***arlS***
**SA112**	**2560**	**Ser-84-Leu, Glu-88-Lys,** Ala-457-Thr	Gln-66-Lys	**Ser-80-Tyr, Glu-84-Lys**, Tyr-410-Phe, **Asp-796-Asn**	Asn-139-Ser	Thr-42-Ala, Thr-166-Met, **Val-189-Ile,** Ser-215-Thr, Phe-278-Val, Thr-318-Ala Leu-352-Ser, Phe-358-Leu, Ser-407-Ala, Ala-417-Val	-	Ser-332-Ile, Ala-364-Thr	-	-	
SA111	**1280**	**Ser-84-Leu,** Ala-457-Thr	Gln-66-Lys	**Ser-80-Tyr, Glu-84-Lys,** Tyr-410-Phe	Asn-139-Ser	Thr-42-Ala, **Leu-140-Ile**, Thr-166-Met, **Val-189-Ile,** Ser-215-Thr, Phe-278-Val, Thr-318-Ala, Leu-352-Ser, Phe-358-Leu, Ser-407-Ala, Ala-417-Val,	-	Ser-332-Ile, Ala-364-Thr	-	-	
**SA113**	**1280**	**Ser-84-Leu**, Ala-457-Thr	Gln-66-Lys	**Ser-80-Tyr, Glu-84-Lys,** Tyr-410-Phe,	Asn-139-Ser	Thr-42-Ala, **Leu-140-Ile**, Thr-166-Met, **Val-189-Ile,** Ser-215-Thr, Phe-278-Val, Thr-318-Ala, Leu-352-Ser, Phe-358-Leu, Ser-407-Ala, Ala-417-Val	**Leu-64-Pro**	Ser-332-Ile, Ala-364-Thr	-	-	

**SA101**	**128**	**Ser-84-Leu,** Asp-495-Asn	-	**Ser-80-Tyr**	-	Phe-278-Val, Ser-407-Ala,	-	-	-	-	
**M43-01**	**128**	**Ser-84-Leu,** **Asn-842-Ser**, **Thr-845-Ala,** **Ile-855-Met**	Gln-66-Lys	**Ser-80-Phe,** Ile-223-Val	-	**Ala-58-Val,** Glu-131-Asp, Ser-160-Thr, Ile-231-Phe, **Phe-237-Ile,** Leu-352-Ser, Phe-278-Val, Thr-318-Ala, Phe-358-Leu, Val-361-Met, Ile-365-Val, Ser-407-Ala	-	**Asp-221-Glu**, **Gly-258-Arg**, Ser-332-Ile	**Ile-16-Val**	**Val-302-Leu**, **Ile-362-Thr**	Glu-121-Asp
**SA107**	**64**	Asp-856-Glu, **Asn-860-Thr**, **Glu-862-Asp**,	Gln-66-Lys	Ile-223-Val, Tyr-410-Phe	**His-478-Tyr**	Thr-42-Ala, Val-106-Ile, Gln-128-Glu, Glu-131-Asp, Ser-160-Thr, Ile-191-Val, Ile-231-Phe, Asn-245-Lys, Phe-278-Val, **Tyr-289-Phe,** Thr-318-Ala, Val-357-Ile, Val-361-Met, Ile-365-Val, Ser-407-Ala, **Leu-412-Ile**	-	Val-167-Ile, Ile-214-Val, Ser-332-Ile, Ala-364-Thr	-	-	
**M5-01**	**64**	**Glu-409-Asp**, Asp-483-Glu	Gln-66-Lys	-	-	**Ile-12-Thr**, Val-106-Ile, Gln-128-Glu, Ser-160-Thr, **Ala-186-Thr**, Ile-191-Val, Ile-231-Phe, Asn-245-Lys, Phe-278-Val, **Ser-321-Leu**, **Ser-331-Thr**, Leu-352-Ser,	-	Val-167-Ile, Ile-214-Val, Ser-332-Ile, Ala-364-Thr	-	-	

						Phe-358-Leu,<br>**Val-394-Ala**,<br>Ser-407-Ala,<br>**Val-423-Ile**					
**SA90**	**64**	**Ser-84-Leu**,<br>Ala-457-Thr	Gln-66-Lys	**Ser-80-Tyr,**<br>**Glu-84-Lys**,<br>Tyr-410-Phe,	Asn-139-Ser	Thr-42-Ala,<br>Thr-166-Met,<br>Ser-215-Thr,<br>Phe-278-Val,<br>Thr-318-Ala,<br>Leu-352-Ser,<br>Phe-358-Leu,<br>Ser-407-Ala,<br>Ala-417-Val	-	Val-167-Ile,<br>Ser-332-Ile	-	-	
**SA102**	**32**	**Ser-84-Leu,**<br>Asp-495-Asn	-	**Ser-80-Phe**,<br>Phe-521-Tyr	-	Phe-278-Val,<br>Ser-407-Ala,	-	-	-	-	
**SA103**	**32**	**Ser-84-Leu**,<br>Asp-495-Asn	-	**Ser-80-Tyr**,<br>**Glu-422-Asp**	-	Phe-278-Val,<br>Ser-407-Ala,	-	-	-	-	
**SA114**	**8**	Asp-402-Glu,<br>Val-598-Ile,<br>Glu-859-Val	Glu-317-<br>Asp	Pro-144-Ser,<br>Phe-521-Tyr,<br>Val-656-Ile	-	Ala-8-Thr,<br>Thr-42-Ala,<br>Gln-128-Glu,<br>Met-159-Val,<br>Ser-160-Thr,<br>**Arg-168-Cys,**<br>Val-173-Gly,<br>Ile-191-Val,<br>Ile-231-Phe,<br>Gly-242-Val,<br>Phe-278-Val,<br>Thr-318-Ala,<br>Leu-329-Phe,<br>Val-339-Ile,<br>Gly-340-Ala,<br>Val-357-Ile,<br>Phe-358-Leu,<br>Val-361-Met,<br>Ile-365-Val,<br>Ser-407-Ala,<br>Tyr-430-Asp	-	Thr-114-Ala,<br>Val-167-Ile,<br>Ala-307-Ser,<br>Ala-364-Thr	Met-15-Ile	-	Glu-121-Asp
**M71-<br>01**	**4**	-	Gln-66-Lys	Tyr-410-Phe,<br>**Leu-450-Phe**	-	Glu-131-Asp,<br>Ser-160-Thr,<br>**Ile-238-Ser**,<br>Asn-245-Lys,	-	Ser-332-Ile	-	**Ala-76-Thr**	**Asp-312-Asn**

						Phe-278-Val,<br>Thr-318-Ala,<br>Val-357-Ile,<br>Phe-358-Leu,<br>Ser-407-Ala,<br>**Val-439-Ile**					
**SA136**	**4**	**Glu-409-Asp**,<br>Asp-483-Glu	Gln-66-Lys	-	-	**Ile-12-Thr**,<br>Val-106-Ile,<br>Gln-128-Glu,<br>Ser-160-Thr,<br>**Ala-186-Thr**,<br>Ile-191-Val,<br>Ile-231-Phe,<br>Asn-245-Lys,<br>Phe-278-Val,<br>**Ser-321-Leu**,<br>**Ser-331-Thr**,<br>Leu-352-Ser,<br>Phe-358-Leu,<br>Ser-407-Ala,<br>**Val-423-Ile**	-	Val-167-Ile,<br>Ile-214-Val,<br>Ser-332-Ile,<br>Ala-364-Thr	-	-	
**SA31**	**4**	Asp-483-Glu,<br>Glu-594-Gly,<br>Glu-697-Lys	-	Pro-144-Ser,<br>Phe-521-Tyr,<br>Val-656-Ile	-	Ala-8-Thr,<br>Val-106-Ile,<br>Gln-128-Glu,<br>Met-159-Val,<br>Ser-160-Thr,<br>Val-173-Gly,<br>Ile-191-Val,<br>Leu-227-Phe,<br>Ile-231-Phe,<br>Gly-242-Val,<br>Asn-245-Lys,<br>Phe-278-Val,<br>Thr-318-Ala,<br>Leu-329-Phe,<br>Gly-340-Ala,<br>Val-357-Ile,<br>Phe-358-Leu,<br>Val-361-Met,<br>Ile-365-Val,<br>Ser-407-Ala,<br>Tyr-430-Asp	-	Thr-114-Ala,<br>Val-167-Ile,	-	-	Glu-121-Asp

**SA86**	**1**	Ala-457-Thr	Gln-66-Lys	Tyr-410-Phe,	Asn-139-Ser	Thr-42-Ala, Thr-166-Met, Ser-215-Thr, Phe-278-Val, Thr-318-Ala, Leu-352-Ser, Phe-358-Leu, Ser-407-Ala, Ala-417-Val	-	Val-167-Ile, Ser-332-Ile	-	-	
**SA34**	**1**	Val-598-Ile, Val-712-Ile, Arg-837-His, Asp-856-Glu	Glu-317-Asp	Ile-45-Met, Ser-267-Gly, Phe-594-Tyr, Ala-688-Val	Glu-596-Asp	Thr-42-Ala, Val-106-Ile, Gln-128-Glu, Glu-131-Asp, Ser-160-Thr, Ile-191-Val, Ile-231-Phe, Pro-264-Ser, Phe-278-Val, Thr-318-Ala, Val-339-Ile, Val-357-Ile, Val-361-Met, Ile-365-Val, Ser-407-Ala, Met-419-Ile, Lys-462-Glu	-	Thr-114-Ala, Val-167-Ile, Ile-214-Val, Val-328-Ile, Ser-332-Ile, Ala-364-Thr	-	-	Glu-121-Asp
**SA129**	**1**	Asp-483-Glu, Glu-594-Gly, Glu-697-Lys	-	Pro-144-Ser, Phe-521-Tyr, Val-656-Ile	Glu-422-Asp	Ala-8-Thr, Thr-42-Ala, Val-106-Ile, Gln-128-Glu, Met-159-Val, Ser-160-Thr, Val-173-Gly, Ile-191-Val, Leu-227-Phe, Ile-231-Phe, Gly-242-Val, Asn-245-Lys, Phe-278-Val, Thr-318-Ala, Leu-329-Phe, Gly-340-Ala,	-	Thr-114-Ala, Val-167-Ile, Ala-307-Ser, Ala-364-Thr	-	-	Glu-121-Asp

						Val-357-Ile,<br>Phe-358-Leu,<br>Val-361-Met,<br>Ile-365-Val,<br>Ser-407-Ala,<br>Tyr-430-Asp					
**M19-01**	**1**	-	Gly-550-Cys	Ile-223-Val	-	Thr-42-Ala,<br>Asn-245-Lys,<br>Phe-278-Val,<br>Thr-318-Ala,<br>Val-361-Met,<br>Val-357-Ile,<br>Ile-365-Val,<br>Ser-407-Ala	-	Val-167-Ile,<br>Ile-214-Val,<br>Gly-269-Arg,<br>Ala-364-Thr,	-	-	
**M28-01**	**1**	-	Gln-66-Lys,<br>Thr-147-Ile,<br>Gln-210-Lys,<br>Asp-589-Asn	-	-	Val-106-Ile,<br>Gln-128-Glu,<br>Ser-160-Thr,<br>Ile-191-Val,<br>Asn-245-Lys,<br>Phe-278-Val,<br>Thr-318-Ala,<br>Val-357-Ile,<br>Phe-358-Leu,<br>Val-361-Met,<br>Ile-365-Val,<br>Ser-407-Ala	-	Tyr-268-His,<br>Ser-332-Ile,<br>Asn-369-Tyr	-	-	
**SA46**	**1**	Ala-457-Thr	Gln-66-Lys	Tyr-410-Phe	Asn-139-Ser	Thr-42-Ala,<br>Thr-166-Met,<br>Ser-215-Thr,<br>Phe-278-Val,<br>Thr-318-Ala,<br>Leu-352-Ser,<br>Phe-358-Leu,<br>Ala-417-Val,<br>Ser-407-Ala	-	Val-167-Ile,<br>Ser-332-Ile	-	-	
**SA20**	**1**	Asp-483-Glu,<br>Glu-594-Gly,	Gln-66-Lys	Val-656-Ile,	Glu-422-Asp,<br>Glu-596-Asp	Thr-42-Ala,<br>Gln-128-Glu,<br>Glu-131-Asp,<br>Ser-190-Asn,<br>Ile-191-Val,<br>His-228-Asn,<br>Ile-231-Phe,	-	Thr-114-Ala,<br>Val-167-Ile,<br>Ile-214-Val,<br>Ala-307-Ser,<br>Val-329-Leu,<br>Ala-364-Thr	-	Val-346-Leu,<br>Ala-424-Val	Glu-121-Asp,<br>Asp-133-Tyr

						Val-238-Ala, Ser-243-Thr, Thr-318-Ala, Val-361-Met, Ile-365-Val, Phe-278-Val, Val-357-Ile, Phe-358-Leu, Ser-407-Ala					
**SA25**	**1**	Ala-457-Thr	Gln-66-Lys	Tyr-410-Phe	Asn-139-Ser	Thr-42-Ala, Thr-166-Met, Ser-215-Thr, Phe-278-Val, Thr-318-Ala, Leu-352-Ser, Phe-358-Leu, Ser-407-Ala, Ala-417-Val	-	Val-167-Ile, Phe-188-Leu, Ser-332-Ile	-	-	-
**SA27**	**1**	Asp-402-Glu, Val-598-Ile, Glu-859-Val	Glu-317-Asp	Pro-144-Ser, Phe-521-Tyr, Val-656-Ile	His-218-Asn	Ala-8-Thr, Thr-42-Ala, Gln-128-Glu, Ser-160-Thr, Val-173-Gly, Ile-191-Val, Ile-231-Phe, Gly-242-Val, Phe-278-Val, Thr-318-Ala, Leu-329-Phe, Val-339-Ile, Gly-340-Ala, Val-357-Ile, Phe-358-Leu, Val-361-Met, Ile-365-Val, Ser-407-Ala, Tyr-430-Asp	-	Thr-114-Ala, Val-167-Ile, Ala-307-Ser, Ala-364-Thr	Met-15-Ile	-	Glu-121-Asp
**SA32**	**1**	Asp-495-Asn	-	-	-	Phe-278-Val, Ser-407-Ala	-	-	-	-	
**SA48**	**1**	Ala-457-Thr	Gln-66-Lys	Tyr-410-Phe	Asn-139-Ser	Thr-42-Ala, Thr-166-Met, Ser-215-Thr,	-	Val-167-Ile, Ser-332-Ile	-	-	

						Phe-278-Val,<br>Thr-318-Ala,<br>Leu-352-Ser,<br>Phe-358-Leu,<br>Ser-407-Ala,<br>Ala-417-Val					

Bold indicates that the changes were found only in resistant isolates; Red indicates likely change in protein function as the result of the change in amino acid sequence.

Supplementary Table S3. Mutations in MMR system of *S. aureus* ocular strains from infections and non-infectious disease group.

***S. aureus* isolates**	**CIP MIC (µg/ml)**	**Total variations**	**Complex**	**Insertions**	**Deletions**	**MNPs**	**SNPs**	**MMR system**	
								mutL	*mutS*
**SA112**	**2560**	39,301	6,224	263	328	1014	31,472	**His-347-Tyr,** Pro-379-Ser, Glu-382-Ala, Ala-409-Thr	Lys-840-Glu, Ser-324-Thr
**SA111**	**1280**	18,320	2,217	154	217	466	15,266	**His-347-Tyr,** Pro-379-Ser, Glu-382-Ala, Ala-409-Thr	Lys-840-Glu, Ser-324-Thr
**SA113**	**1280**	18,157	2,193	150	222	455	15,137	**His-347-Tyr,** Pro-379-Ser, Glu-382-Ala, Ala-409-Thr	Lys-840-Glu, Ser-324-Thr
**SA101**	**128**	18,049	2,208	148	216	458	15,019	0	Lys-840-Glu
**M43-01**	**128**	18,025	2,136	154	226	426	15,083	Asn-361-Ser, Gly-446-Asp	Gln-416-Arg, Lys-840-Glu
**SA107**	**64**	17,647	2,159	146	212	432	14,698	Pro-379-Ser, Glu-382-Ala, Ala-409-Thr	Lys-840-Glu, Thr-142-Ala, Ala-229-Val
**M5-01**	**64**	15,867	1,919	114	183	388	13,263	Lys-108-Asn, Lys-392-Arg, Glu-382-Val,	Lys-840-Glu, Arg-310-Pro

								Thr-448-Ser	
**SA90**	**64**	2,339	337	33	71	47	1,851	**His-347-Tyr,** Pro-379-Ser, Glu-382-Ala, Ala-409-Thr	Lys-840-Glu, Ser-324-Thr
**SA102**	**32**	2,267	322	33	70	49	1,793	0	Lys-840-Glu
**SA103**	**32**	1,367	136	37	68	27	1,099	0	Lys-840-Glu
**SA114**	**8**	44,256	7,904	275	317	1,246	34,517	Asn-365-Lys, Glu-382-Ala, Thr-448-Ser, Val-528-Ile	Asn-181-His, Lys-840-Glu, Glu-66-Asp, Gln-157-His, Met-287-Ile, Val-239-Ala, Asn-373-Asp, Thr-415-Met, **Gln-531-His,** Leu-811-Ser
**M71-01**	**4**	40,332	6,428	270	330	1,105	32,199	Asp-337-Gly, Lys-392-Arg, Glu-382-Val, Thr-448-Ser, His-347-Tyr	Lys-840-Glu, Gln-157-His, Ile-193-Leu

**SA136**	**4**	18,610	2,930	160	181	426	14,913	Lys-392-Arg, Thr-448-Ser	Lys-840-Glu, Thr-142-Ala, Ala-229-Val
**SA31**	**4**	18,139	2,802	164	193	438	14,542	Asn-365-Lys, Glu-382-Ala, Thr-448-Ser, Val-528-Ile	Asn-181-His, Lys-840-Glu, Leu-16-Ile, Glu-66-Asp, Gln-157-His, Met-287-Ile, Val-239-Ala, Asn-373-Asp, Thr-415-Met, **Gln-531-His,** Leu-811-Ser
**SA86**	**1**	17,879	2,221	155	224	448	14,831	**His-347-Tyr,** Pro-379-Ser, Glu-382-Ala, Ala-409-Thr	Gln-136-His, Asp-300-Glu, Lys-840-Glu, Ser-324-Thr
**SA34**	**1**	16,238	2,054	149	204	401	13,430	Asn-364-Asp, Ser-377-Arg,	Asn-373-Asp,

								Glu-382-Ala, Lys-392-Arg, Asn-418-Asp	Thr-415-Met, Phe-122-Ile, **Ala-172-Val,** Glu-332-Asp, Pro-425-Ser, Leu-811-Ser, Ser-814-Cys, Lys-840-Glu, Glu-66-Asp
**SA129**	**1**	15,944	1,938	149	185	390	13,282	Asn-181-His, Val-239-Ala, Asn-373-Asp, Thr-415-Met, Gln-531-His, Leu-811-Ser, Lys-840-Glu, Leu-16-Ile, Glu-66-Asp	Asn-365-Lys, Glu-382-Ala, Thr-448-Ser, Val-528-Ile
**M19-01**	**1**	15,762	1,883	129	177	389	13,184	Glu-382-Val, Lys-392-Arg, Thr-448-Ser, **Val-583-Ile**	Lys-840-Glu, Arg-310-Pro

**M28-01**	**1**	15,077	1,951	130	182	365	12,449	Glu-382-Val, Lys-392-Arg, Thr-448-Ser, **Val-583-Ile**	Lys-840-Glu, Leu-16-Ile, Glu-66-Asp, Gln-157-His, Cys-189-Tyr
**SA46**	**1**	40,473	6,495	264	330	1,127	32,257	Pro-379-Ser, Glu-382-Ala, Ala-409-Thr, Glu-382-Val	Lys-840-Glu, Ser-324-Thr
**SA20**	**1**	39,481	6,236	258	325	1,086	31,576	Glu-382-Ala, Thr-448-Ser	Asn-181-His, Thr-443-Ile, Asn-373-Asp, Thr-114-Ser, Thr-415-Met, **Gln-531-His,** Leu-811-Ser, Lys-840-Glu, Leu-16-Ile, Glu-66-Asp, Gly-789-Asp

**SA25**	**1**	33,525	4,642	270	328	895	27,390	**His-347-Tyr,** Pro-379-Ser, Glu-382-Ala, Ala-409-Thr	Lys-840-Glu, Ser-324-Thr
**SA27**	**1**	17,700	2,147	144	210	447	14,752	Glu-382-Ala, Thr-448-Ser, Val-528-Ile	Asn-181-His, Lys-840-Glu, Leu-16-Ile, Glu-66-Asp, Gln-157-His, Met-287-Ile, Val-239-Ala, Asn-373-Asp, Thr-415-Met, **Gln-531-His,** Leu-811-Ser
**SA32**	**1**	17,435	2,127	146	222	224	14,495	0	Lys-840-Glu, Ser-364-Ile
**SA48**	**1**	847	29	39	56	4	719	**His-347-Tyr,** Pro-379-Ser, Glu-382-Ala, Ala-409-Thr	Lys-840-Glu, Ser-364-Ile

Bold = presumed functional effect of the protein
